# Effects of household water-repellent agents and number of coating layers on the physical properties of cotton woven fabrics

**DOI:** 10.1371/journal.pone.0283261

**Published:** 2023-04-14

**Authors:** Seyeon Kim, Jo-Eun Kim, Da-Eun Song, Soo-Yeon Cho, Yeseul Hwang, Youngjoo Chae

**Affiliations:** Department of Clothing & Textiles, Chungbuk National University, Cheongju, South Korea; University of Queensland, AUSTRALIA

## Abstract

The increased interest in outdoor activities has prompted the demand for water-repellent fabrics that can withstand various environmental factors. In this study, the water repellency and physical properties, namely thickness, weight, tensile strength, elongation, and stiffness, of cotton woven fabrics were analyzed according to various treatments with different types of household water-repellent agents and number of coating layers. Fluorine-, silicone-, and wax-based water-repellent agents were coated on cotton woven fabrics once, thrice, and five times. Thickness, weight, and stiffness increased with the number of coating layers, which may reduce comfort. These properties increased minimally for the fluorine- and silicone-based water-repellent agents, whereas they considerably increased for the wax-based water-repellent agent. The fluorine-based water-repellent agent had a low water repellency rating of 2.2 even after five coating layers, and the silicone-based water-repellent agent had a higher rating of 3.4 with the same five coating layers. Meanwhile, the wax-based water-repellent agent had the highest water repellency rating of 5 even with only one coating layer, which was maintained with repeated coatings. Therefore, fluorine- and silicone-based water-repellent agents minimally altered the fabric properties even with repeated coatings; multiple coating layers, especially five or more layers for the fluorine-based water-repellent agent, are recommended to attain excellent water repellency. Conversely, one coating layer of the wax-based water-repellent agent is recommended to retain the comfort of the wearer.

## Introduction

The rapid global proliferation of COVID-19 has prompted the implementation of social distancing in different countries. The prolonged pandemic owing to the continuous mutations has increased the demand for short-distance domestic travels rather than overseas travels. Consequently, more people are enjoying outdoor activities, such as camping and hiking. To accommodate this broad range of consumers, outdoor merchandise brands are striving to develop products with excellent design and performance. In particular, outdoor clothing must be equipped with water repellency to protect the wearer from rain and other outdoor liquid pollutants. Water repellency prevents water from permeating the surface of a fabric with low surface tension [1]. It is realized by applying and attaching a hydrophobic water-repellent agent to fabrics using low-temperature plasma pretreatment or a pad-dry-cure method without pretreatment [2]. Recently, an increasing number of consumers are demanding water repellency not only in outdoor clothes but also in everyday clothes to prevent contamination from liquid substances, such as rain, beverages, and splashes.

Water-repellent agents are hydrophobic and oil-repellent, thereby endowing the products with self-cleaning, oil/water separation, and fluid drag reduction properties, among others [3]. Silicone- and fluorine-based agents are examples of water-repellent agents used in textiles. Fluorine-based water-repellent agents have been used in various industries and day-to-day items for decades because of their excellent water repellency owing to the strong bonding strength between the fluorine atoms and carbon, and low polarizability. Moreover, they are oil-repellent and soil-proof because of their chemical stability and low surface energy [4, 5]. However, the fluorine-based water- repellent agents are expensive, have low durability, and are currently restricted worldwide owing to their potential environmental hazards and biotoxicity [2]. Thus, silicone, wax, paraffin, polyurethane, and dendrimers are used as alternatives to fluorine-based water-repellent agents [3]. Among them, silicone-based water-repellent agents are prepared by introducing a siloxane polymer or a long chain fatty acid into a silicone-based material. Since they have a stable chemical structure, no oxidation and corrosion, and excellent water repellency, thermal stability and washing durability, they have been widely used as a substitute to fluorine-based water-repellent agents [4, 6]. Meanwhile, wax-based water-repellent agents are prepared by dissolving wax onto the product through heating, thus imparting hydrophobicity to the product facilely. However, wax-based water-repellent agents contain harmful ingredients; they are difficult to apply evenly, require a heat treatment process, and can stiffen clothes [7]. Moreover, they cause lower ventilation, which is an important sub-attribute of garments that affects overall comfort, and can easily be removed during washing or dry cleaning. Therefore, various attempts have been made recently, such as producing colloidal wax particles, to overcome these limitations of wax-based water-repellent agents [7].

Previous research has been conducted on the application of water-repellent agents and changes in the physical properties of fabrics according to the treatment conditions. Yu et al. [8] analyzed the changes in washing fastness in water repellency of nylon fabrics with different treatment concentrations of fluorine-based water-repellent agents and addition of a crosslinking agent. Choi et al. [2] compared the performances of general fluorine- and nano-sized fluorine-based water-repellent agents. The results showed that the fabrics treated with nano-sized fluorine-based water-repellent agents had superior water and oil repellency, wrinkle recovery rates, and washing fastness in water repellency compared to the fabrics treated with general fluorine-based water-repellent agents. Lee et al. [9] evaluated the differences in water repellency of polyester fabrics according to the treatment concentration and curing temperature of a fluorine-based water-repellent agent and the electron beam irradiation pretreatment during the processing. The results showed the increase in water repellency with the concentration of the water-repellent agent when the fabric was irradiated with electron beam during the pretreatment. Meanwhile, Koo et al. [10] compared the water repellency, moisture permeability, stiffness, crease resistance, and washing fastness of the cotton, nylon, wool/polyester, and cotton/polyester fabrics treated with polyurethane-, acrylic-, and silicone-based water-repellent agents. Among different types of fabrics, nylon fabrics exhibited the best water repellency and crease resistance after water-repellent treatment. Also, regardless of the fabric type, the fabrics treated with the polyurethane-based water-repellent agent had the best water repellency and crease resistance, but were stiffer than the fabrics treated with the acrylic-based water-repellent agent. In other relevant studies done by Mazzon et al. [11, 12], cotton fabrics were treated with a polyurethane modified aminosilicone fluid and an acetoxy functional biocompatible hydrophobic silicone to impart hydrophobicity. Despite the thorough analysis of water-repellent treatment under systematic experimental designs, most of these previous studies evaluated only one water-repellent agent. Also, they have not considered easy application to clothing products for actual daily wear by focusing on industrial rather than household water-repellent agents.

For everyday clothes using household water-repellent agents, comfort is as important as the water repellency of the material. Hence, various physical sub-attributes should be considered. Furthermore, the changes in the physical properties of the fabric according to treatment with various water-repellent agents should be investigated. This study analyzed the physical properties of cotton woven fabrics according to the type of water-repellent agent used for treatment, and number of coating layers using commercially available fluorine-, silicone-, and wax-based water-repellent agents. The optimal water repellency treatment conditions for clothing products were determined. The specific research objectives are as follows. First, the differences in the thickness, weight, tensile strength, elongation, stiffness, and water repellency, which determine the overall comfort of the fabric, according to the type of water-repellent agents used in treatment were analyzed. Second, the changes in these properties according to the number of coating layers were compared.

## Methodology

### Fabric sample

Commercially available untreated cotton woven fabrics were used as samples for the treatment of household water-repellent agents. Cotton woven fabric was used because cotton is the most commonly used textile fiber, alone or mixed, in various clothing products. In addition, cotton is hydrophilic and not inherently water-repellent, making it suitable for analyzing the effects of treatment with water-repellent agents. The samples were 100% cotton plain woven fabrics without any other fiber mixed in, with a yarn density of 32×29/in^2^ and thickness of 0.34 mm. Table 1 summarizes the structural characteristics of the samples used in this study.

**Table 1:** Specifications of the fabric sample (Substrate).

Material	Fabric type	Weave	Yarn density (warp × weft/in^2^)	Fabric thickness (mm)	Color
100% cotton	Woven	Plain	32**×**29	0.34	Unbleached off-white

### Water-repellent agents and treatment methods

Fluorine-based water-repellent agents (Fabric crystal, Sensha, Japan), silicone-based water-repellent agents (Gore-king, SnailFix, Korea), and wax-based water-repellent agents (Waxwing original reform wax, Yubong, Korea) were used as the household water-repellent agents in this study. The cotton woven fabric sample was coated once, three times, and five times, respectively, according to the methods presented in the instructions for use of each water-repellent agent. The fluorine- and silicone-based water-repellent agents in liquid state were transferred to the same empty spray bottle and sprayed on the sample at a distance of 15 cm to apply the same amount per coating. After spraying, the samples were cured at 80°C for 10 min. The wax-based water-repellent agent in solid state was coated on the sample at 0.0133 g/cm^2^ per sample area and the sample cured at 80°C for 10 min after each coating. For multiple coatings, the aforementioned process was repeated three and five times, respectively. A total of 10 samples, including nine samples treated with water-repellent agents (three types of water-repellent agents × three coating conditions), and one untreated sample were prepared for the evaluation of physical properties. Table 2 summarizes the details of the samples.

**Table 2:** Samples prepared for the evaluation of the physical properties.

Sample name	Water-repellent agent	Number of coating layers
UT	–	0 (Untreated)
F-1	Fluorine-based	1
F-3	Fluorine-based	3
F-5	Fluorine-based	5
S-1	Silicone-based	1
S-3	Silicone-based	3
S-5	Silicone-based	5
W-1	Wax-based	1
W-3	Wax-based	3
W-5	Wax-based	5

### Evaluation of the physical properties

The physical properties of the samples were measured five times each in a standard laboratory environment (temperature 20±2°C, relative humidity 65±2%). The thickness, weight, tensile strength, elongation, stiffness, and water repellency, which are important in determining the overall clothing comfort of various clothing products, including outdoor clothing, were measured. Before each measurement, all the samples were kept in the standard state for at least 24 h until they reached moisture equilibrium.

#### Thickness

The sample thickness was measured using a thickness gauge (Mitutoyo, Japan) in accordance with International Organization for Standardization (ISO) 5084:1996 [13]. A sample in the standard state was placed on the reference plate of the thickness-measuring device, and a constant load was applied using a presser foot. The number on the dial, which is the vertical distance from the reference plate, was read after 5 s.

#### Weight

In addition to the thickness, tensile properties, and stiffness, the weight of the fabric determines the clothing pressure and is a major factor that affects the motor functional comfort of the wearer. The weight of a 10 cm×10 cm (length × width) sample was measured using an electronic scale (GF-300, AND, Japan). Based on this measurement, the mass per unit area was calculated using Eq (1), as suggested in ISO 3801:1977 [14]:

$$m_{ua}=\frac{m_{c}}{l_{c}\times w_{c}}$$ (1)

where *m*_ua_ is the mass per unit area, in g/m^2^; *m*_c_ is the mass, in g; *l*_c_ is the length, in m; and *w*_c_ is the width, in m, of the sample after conditioning in the standard atmosphere for testing.

#### Tensile properties

The tensile strength and elongation of the samples were measured in the warp direction using a tensile-testing machine (HS-302A, Han Won Soway Co., Republic of Korea), according to the strip method suggested in ISO 13934–1:2013 [15]. A 5 cm × 25 cm (width × length) sample with a gauge length of 20 cm was clamped to the tensile-testing machine. Considering the mass per unit area (m_ua_) of the sample, an approximate pretension (≤200 g/m^2^: 2 N; 200–500 g/ m^2^: 5 N; >500 g/m^2^: 10 N) was applied. The rate of extension was set to 100 mm/min. The tensile strength was presented by the maximum force recorded when the sample ruptured. The elongation at this maximum force was also obtained. The percent elongation was calculated using Eq (2) based on the original and extended length of the sample:

$$\%\delta =\frac{L_{f}-L_{o}}{L_{o}}x\;100$$ (2)

where %*δ* is the percent elongation, that is, the ratio of the extension of the sample to its initial length; *L*_f_ is the final sample length, in cm; and *L*_o_ is the original sample length, in cm.

#### Stiffness

The stiffness of the samples was measured in the warp direction using a fabric stiffness tester (Refond Equipment Co., RF3302, Hong Kong), according to the method suggested by the American Society for Testing and Materials (ASTM) D1388 [16]. After one end of a 15 cm×2.5 cm (length × width) sample was placed on the 0 mark on the scale ruler of the stability tester, the sample was slowly pushed to the slope plane until its opposite end reached a slope plane of 41.5°. The pushed length (cm) was measured. The drape stiffness, which represents the movement resistance of the fabric, and flex stiffness, which represents the resistance of the fabric to flexural deformation, were calculated using Eqs (3) and (4), respectively, based on the pushed length and weight of the sample.

C=D/2(cm) (3)

where *C* is the drape stiffness of the sample and *D* is the pushed length of the sample, in cm.

$$E=C^{3}W\;(cm∙g)$$ (4)

where *E* is the flex stiffness of the sample and *W* is the weight of the sample, in g/cm^2^.

#### Water repellency

The water repellency of the samples was evaluated using the spray test method suggested in ISO 4920:2012 [17]. A 20 cm×20 cm (length × width) sample was fixed at an angle of 45° and at a distance of 15 cm from the nozzle, from which water was discharged from the spray device. Subsequently, 250 mL (27±2°C) distilled water was dropped into a funnel connected to the nozzle for 30 s. After the water was completely dripped, the excess water droplets on the sample were removed gently shaking. The degree of wetting on the face of the sample, from which the droplets were removed, was compared with the descriptive standards and photographs to determine a rating of 1 to 5.

### Data analysis

Statistical analysis was performed using the SPSS program for the physical properties of the cotton woven fabrics treated with water-repellent agents with numerical values (thickness, weight, tensile strength, elongation, and stiffness) and rating (water repellency). First, the Pearson’s correlation analysis was performed using the type of water-repellent agent and number of coating layers as the independent variables, and the evaluated values of the physical properties of the treated fabrics as the dependent variables. The differences in the evaluated values of the physical properties based on the type of water-repellent agent as the specific relationships among significant variables were analyzed by performing a one-way analysis of variance (ANOVA) with Tukey’s post-hoc test. Moreover, the differences in the physical properties according to the number of coating layers were analyzed by performing a simple regression analysis.

## Results and discussion

### Correlation between the type of water-repellent agent and number of coating layers and the physical properties of the treated cotton woven fabrics

Correlation analysis was performed to examine the significance of the relationship between the type of water-repellent agents (independent variable 1: fluorine-, silicone-, and wax-based) and number of coating layers (independent variable 2: 0, 1, 3, and 5 layers), and the physical properties of the treated cotton woven fabric (independent variables: thickness, weight, tensile strength, elongation, drape stiffness, flex stiffness, and water repellency). Table 3 presents the derived correlation coefficients between the variables. The type of water-repellent agent caused a significant difference (*P* < 0.01) in the physical properties of the treated cotton woven fabric, except for the tensile strength and elongation. Similarly, the number of coating layers of the water-repellent agent significantly changed (*P* < 0.01) all the physical properties of the cotton woven fabric, except for the tensile strength.

**Table 3:** Pearson’s correlation coefficients of the studied variables.

Independent variables	Dependent variables						
	Thickness	Weight	Tensile strength	Tensile elongation	Drape stiffness	Flex stiffness	Water repellency
Type of Water-Repellent Agent ^a^	0.420**	0.607**	-0.41	-0.233	0.676**	0.602**	0.653**
Number of coating layers ^b^	0.577**	0.413**	0.196	0.607**	0.444**	0.435**	0.523**

^a^Types of water-repellent agent: fluorine-, silicone-, and wax-based.

^b^ Number of coating layers: 0 (Untreated), 1, 3, and 5.

**P < 0.01

### Changes in the physical properties of the treated cotton woven fabrics according to the type of water-repellent agent

#### Effects of the type of water-repellent agent on thickness

**Fig 1:**
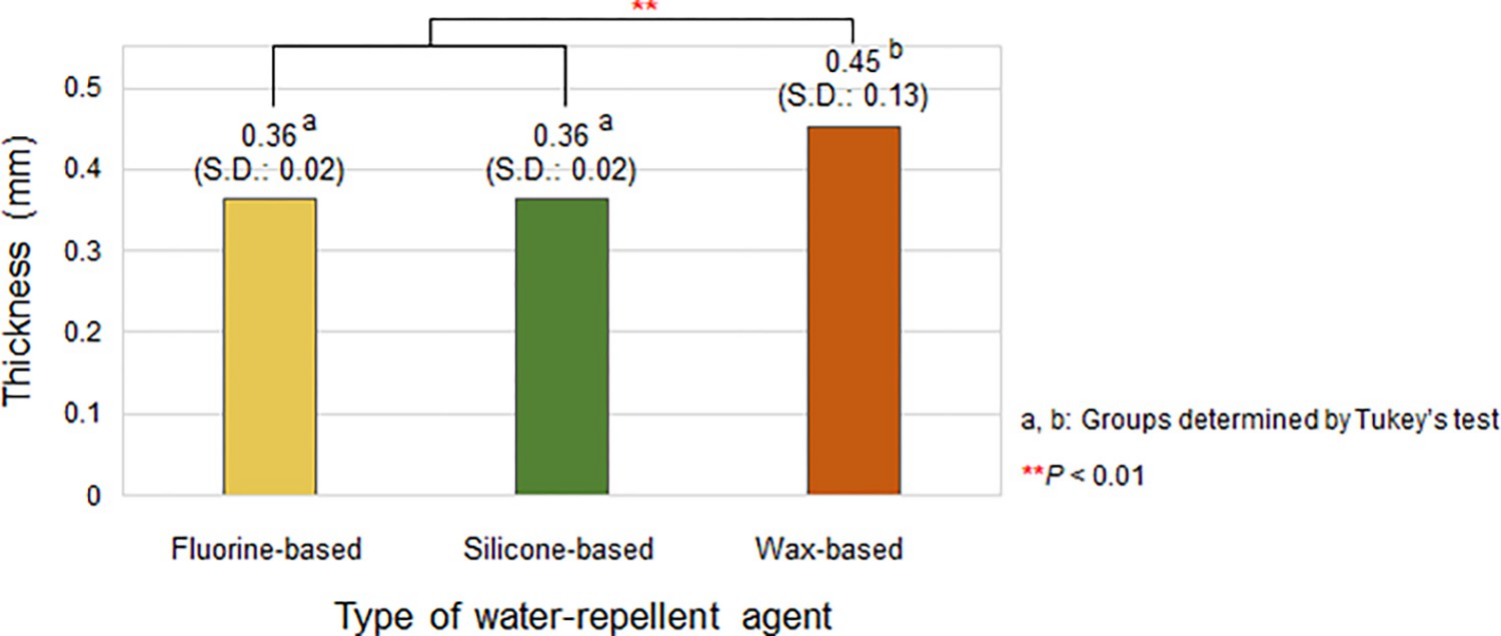
Thickness of the cotton woven fabric according to the type of water-repellent agent. The result is the average thickness of the fabrics of all numbers of coating layers under each water-repellent agent condition.

The average thickness of the treated cotton woven fabrics based on the type of water-repellent agent is presented in Fig 1. The average thickness of the cotton woven fabrics treated with the wax-based water-repellent agent was 0.45 mm (S.D.: 0.13), and that treated with the fluorine- and silicone-based water-repellent agents were identical at 0.36 mm (S.D.: 0.02). Thus, the wax-based water-repellent agent increased the thickness of the treated fabric more than that of the other two water-repellent agents. Compared with the original thickness of 0.34 mm (Table 1) of the untreated cotton woven fabric, the fluorine- and silicone-based water-repellent agents did not significantly change the overall thickness of the treated fabric, resulting in the comfort of the wearer even with repeated coating.

#### Effects of the type of water-repellent agent on weight

**Fig 2:**
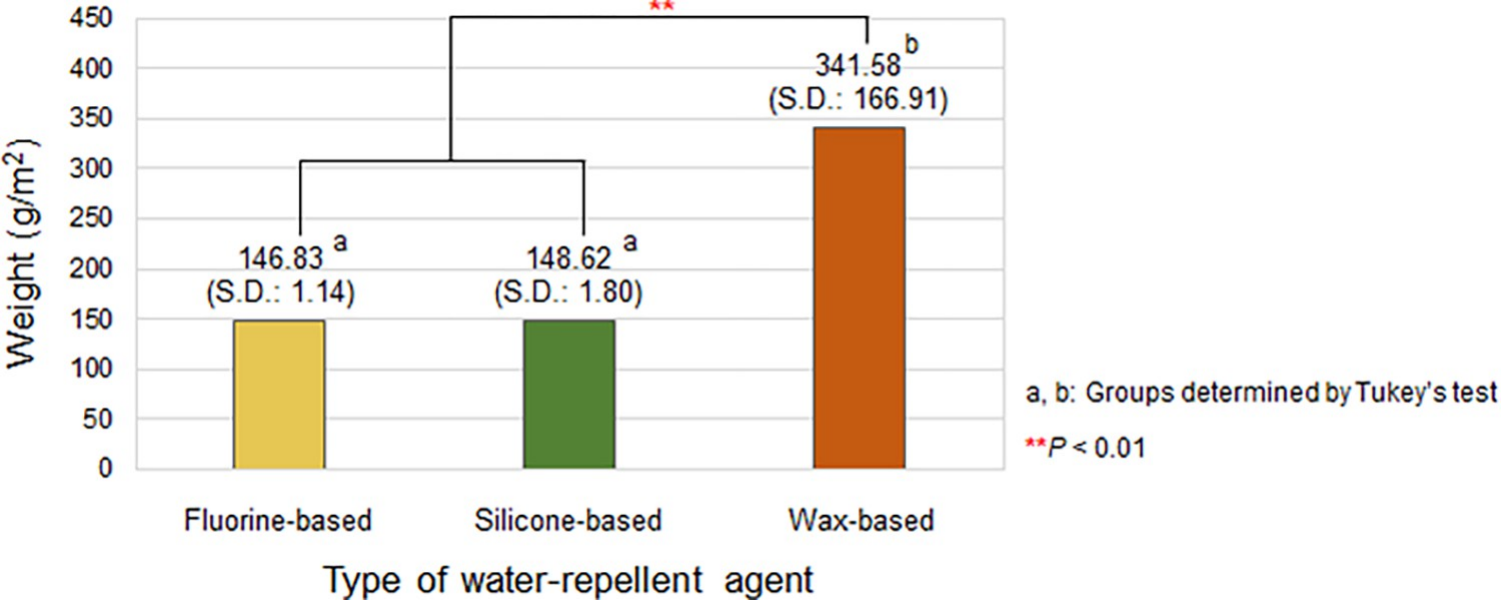
Weight of the cotton woven fabric according to the type of water-repellent agent. The result is the average weight of the fabrics of all numbers of coating layers under each water-repellent agent condition.

Fig 2 presents the results of the one-way ANOVA and post-hoc test on the weights of the cotton woven fabrics based on the types of water-repellent agent used in the fabric treatment. The average weights of the cotton woven fabrics treated with the fluorine- and silicone-based water-repellent agents were 146.83 g/m^2^ (S.D.: 1.14) and 148.62 g/m^2^ (S.D.: 1.80), respectively, which were significantly lower than the average weight of 341.58 g/ m^2^ (S.D.: 166.91) of the cotton woven fabrics treated with the wax-based water repellent. Even when compared with the original weight of 146.80 g/m^2^ of the untreated cotton woven fabric, the fluorine- and silicone-based water-repellent agents barely changed the overall weight of the treated fabric. Meanwhile, the high standard deviation of the weight of the cotton woven fabrics treated with the wax-based water-repellent agent indicates that the increase in weight according to the number of coating layers is significantly higher than that of the other two water-repellent agents, thereby reducing the comfort of the wearer.

#### Effects of the type of water-repellent agent on stiffness

**Fig 3:**
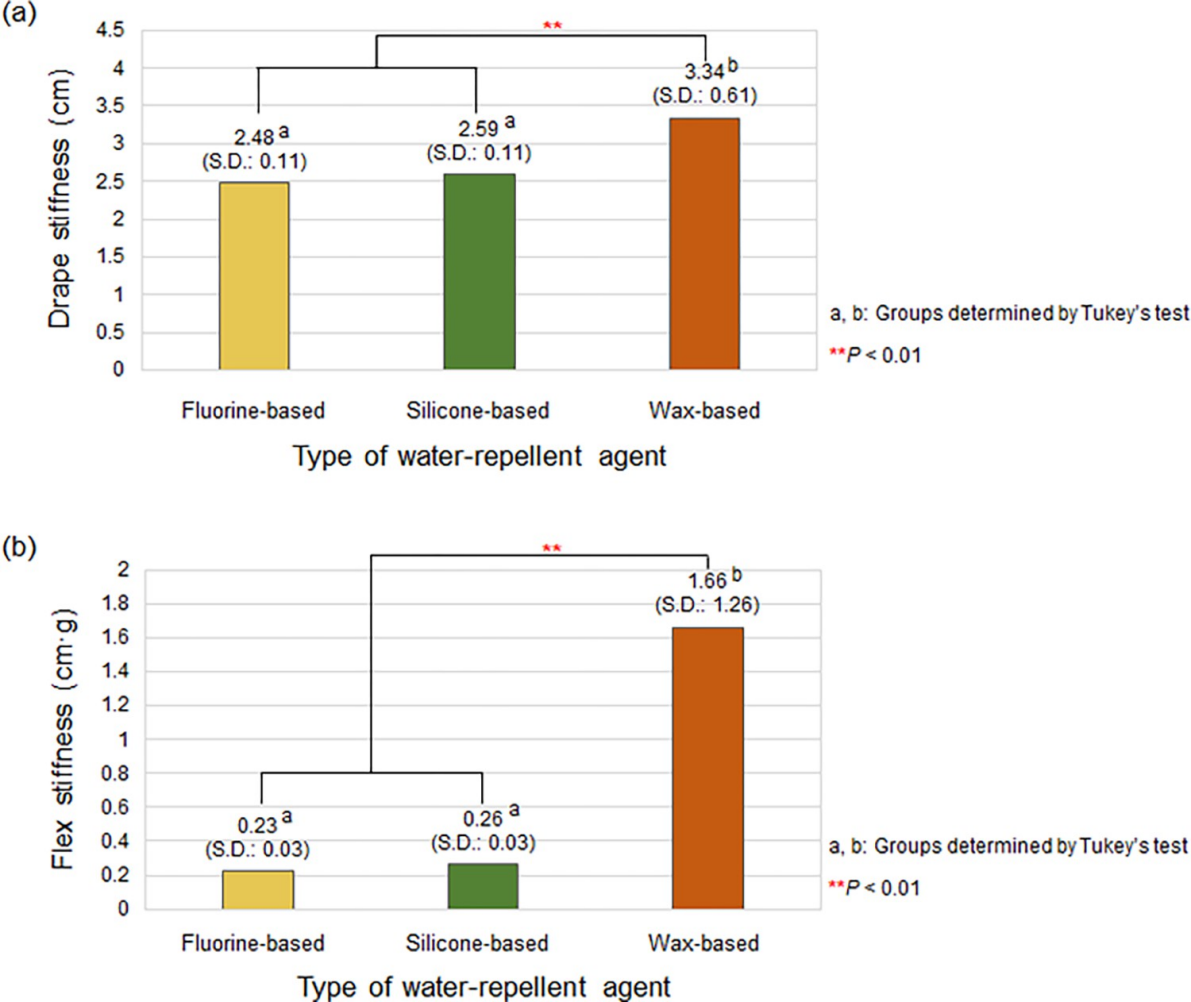
Stiffness of the cotton woven fabric according to the type of water-repellent agent: (a) drape stiffness and (b) flex stiffness. The result is the average stiffness of the fabrics of all numbers of coating layers under each water-repellent agent condition.

Fig 3 presents the stiffness of the treated cotton woven fabrics based on the type of water-repellent agent. The average drape stiffness for the cotton woven fabrics treated with the fluorine-, silicone-, and wax-based water-repellent agents were 2.48 cm (S.D.: 0.11), 2.59 cm (S.D.: 0.11), and 3.34 cm (S.D.: 0.61), respectively, as shown in Fig 3(A). The flex stiffness exhibited the same tendency as that of the drape stiffness, as shown in Fig 3(B), with the highest value of 1.66 cm·g (S.D.: 1.26) for the sample treated with the wax-based water repellent, followed by the sample treated with the silicone-based water-repellent agent (mean: 0.26 cm·g; S.D.: 0.03) and fluorine-based water-repellent agent (mean: 0.23 cm·g; S.D.: 0.03). According to the post-hoc test results for the drape and flex stiffness, the cotton woven fabrics treated with the fluorine- and silicone-based water-repellent agents are in the same group, whereas those treated with the wax-based water-repellent agent are in another group. In particular, the fabrics treated with the fluorine- and silicone-based water-repellent agents were less stiff than those treated with the wax-based water-repellent agent. Even when compared with the original drape stiffness and flex stiffness of 2.41 cm and 0.21 cm·g, respectively, of the untreated cotton woven fabric, the fluorine- and silicone-based water-repellent agents minimally increased the overall stiffness of the treated fabric. The reason for the higher stiffness of the fabrics treated with the wax-based water-repellent agent is that the wax-based water-repellent agent in solid state has large particle size and thus a considerable amount of the agent is thickly deposited onto the fabric surface in a solid state. The significant changes in stiffness, together with those in thickness and weight, caused by the wax-based water-repellent agent suggest that the wax-based agent may reduce the comfort of the wearer in performing motor functions compared to the other two water-repellent agents in liquid state.

#### Effects of the type of water-repellent agent on water repellency

**Fig 4:**
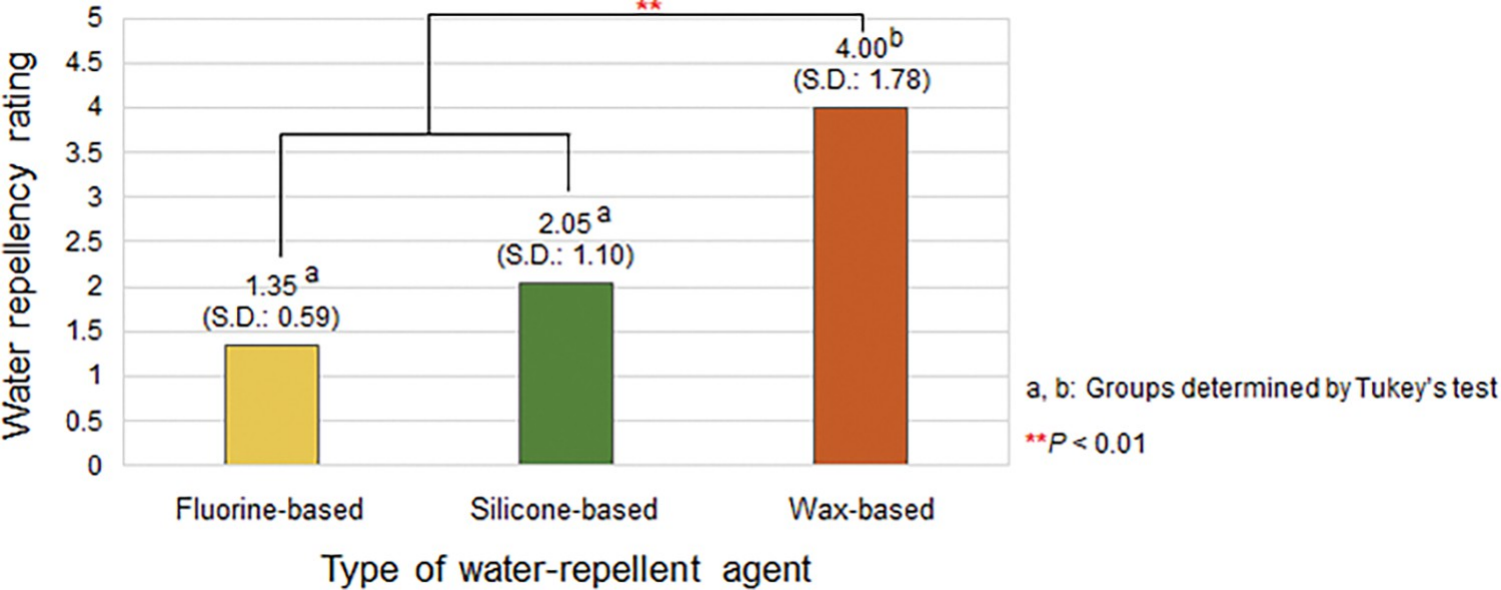
Water repellency of the cotton woven fabric according to the type of water-repellent agent. The result is the average rating of the fabrics of all numbers of coating layers under each water-repellent agent condition.

Fig 4 presents the average water repellency ratings of the cotton woven fabrics based on the type of water-repellent agents used to treat the fabrics. The average water repellency rating of the cotton woven fabrics treated with the fluorine- and silicone-based water-repellent agents were 1.35 (S.D.: 0.59) and 2.05 (S.D.: 1.10), respectively, which were significantly lower than the average water repellency rating of 4 (S.D.: 1.78) of cotton woven fabrics treated with the wax-based water-repellent agent. According to the post-hoc test, similar to the other physical properties, the cotton woven fabrics treated with the fluorine- and silicone-based water-repellent agents were grouped into one group, whereas those treated with the wax-based water-repellent agent were grouped into another. Thus, the fluorine- and silicone-based water-repellent agents in liquid state endowed the treated fabrics with similar physical and functional properties, unlike the solid wax-based water-repellent agent. Based on water repellency only, the wax-based water-repellent agent was the most effective among the three water-repellent agents. However, a water-repellent agent should be selected considering the preferred physical properties, including the overall comfort of the wearer. For example, when considering low weight gain and yet still good water repellency, the silicone-based water-repellent agent will be a better option.

### Changes in the physical properties of the treated cotton woven fabrics according to the number of coating layers of the water-repellent agent

#### Changes in the thickness according to the number of coating layers of the water-repellent agent

**Fig 5:**
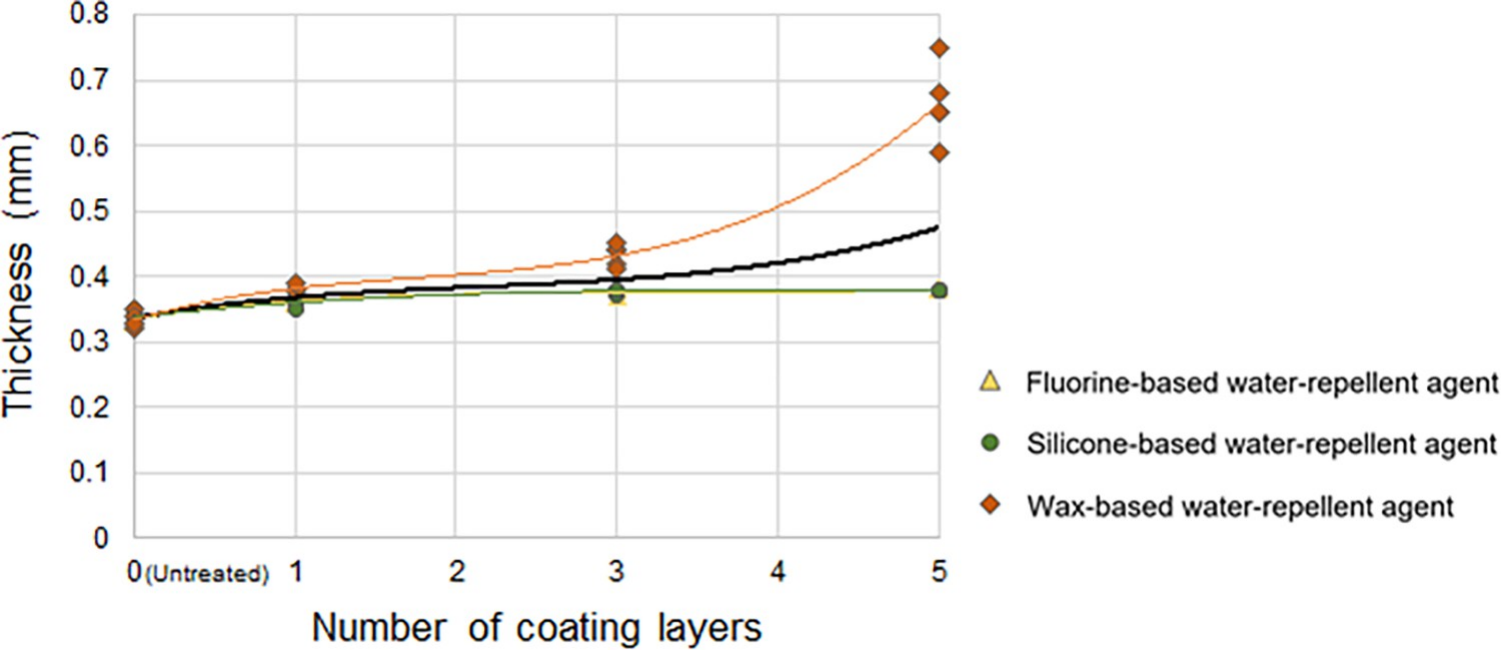
Thickness of the cotton woven fabric according to the number of coating layers of the water-repellent agent. The black line represents the trendline (third-order polynomial).

Fig 5 presents the changes in the thickness of the treated cotton woven fabrics according to the number of coating layers of the water-repellent agents. For each water-repellent agent, the thickness of the treated cotton woven fabrics increased as the number of coating layers increased for all water-repellent agents. For the wax-based water-repellent agent, the thickness of the treated fabric rapidly increased as the number of coating layers increased, by which the average thickness of fabrics was approximately twice the original thickness of the fabrics with five coating layers (0.66 mm). Conversely, the liquid fluorine- and silicone-based water-repellent agents increased the thickness of the fabrics by an average of 0.02 mm from their original thickness with one coating layer, and the thickness with one coating layer (0.36 mm) was maintained even with repeated coatings (average of 0.38 mm for five coating layers).

#### Changes in the weight according to the number of coating layers of the water-repellent agent

**Fig 6:**
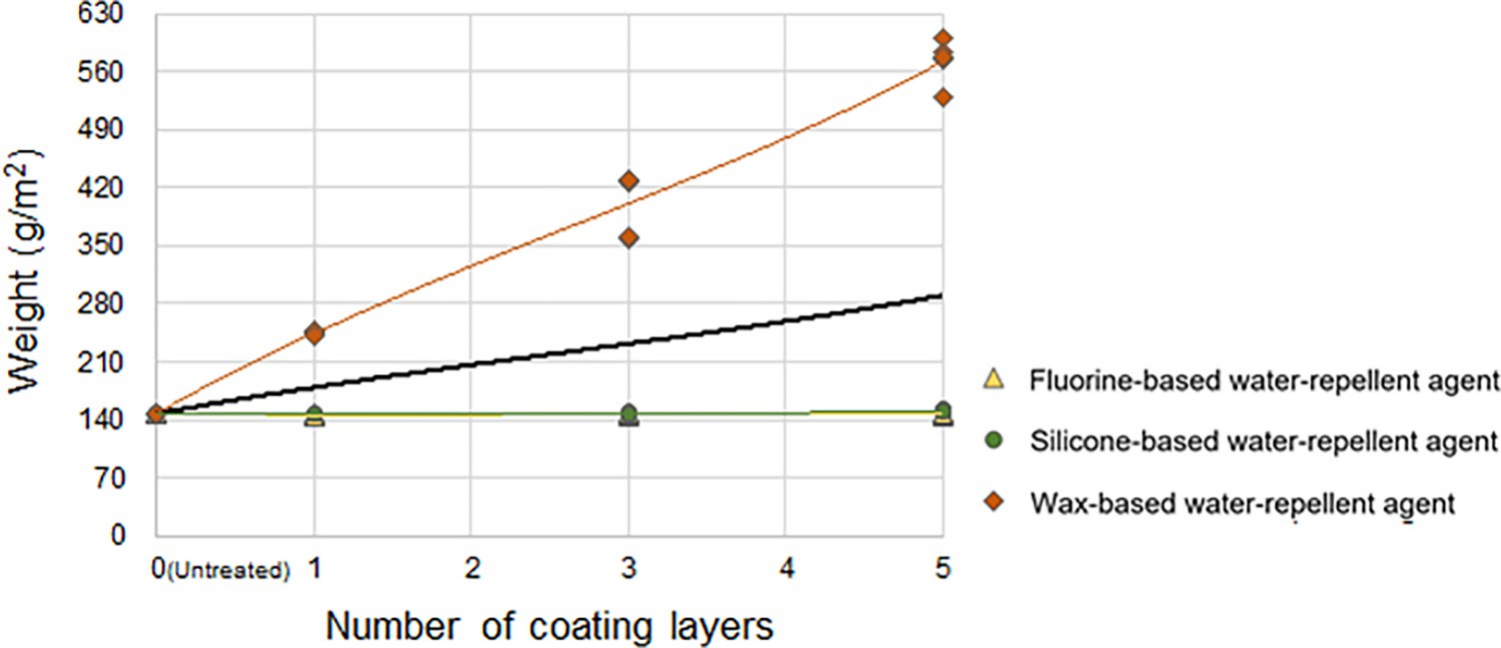
Weight of the cotton woven fabric according to the number of coating layers of the water-repellent agent. The black line represents the trendline (third-order polynomial).

Fig 6 presents the changes in the weights of the treated cotton woven fabrics according to the number of coating layers of the water-repellent agents. The fluorine- and silicone-based water-repellent agents did not significantly change the original weights of the treated fabrics even with the increased number of coating layers (average with no treatment: 146.80 g/m^2^; average with five coating layers of the fluorine- and silicone-based water-repellent agent: 146.84 and 151.24 g/m^2^, respectively). Conversely, for the wax-based water-repellent agent, the weight of the treated fabric rapidly increased with the number of coating layers: 244.62, 401.48, and 572.76 g/m^2^ with one, three, and five coating layers, respectively. This result is attributed to the adsorption of most of the liquid fluorine- and silicone-based water-repellent agents into the cotton woven fabric even with repeated coatings due to their low viscosity and small particle size, whereas only a certain amount of the wax-based water-repellent agent with much higher viscosity and larger particle size is absorbed by the cotton woven fabric and a considerable amount is deposited onto the surface in a solid state.

#### Changes in the tensile elongation according to the number of coating layers of the water-repellent agent

**Fig 7:**
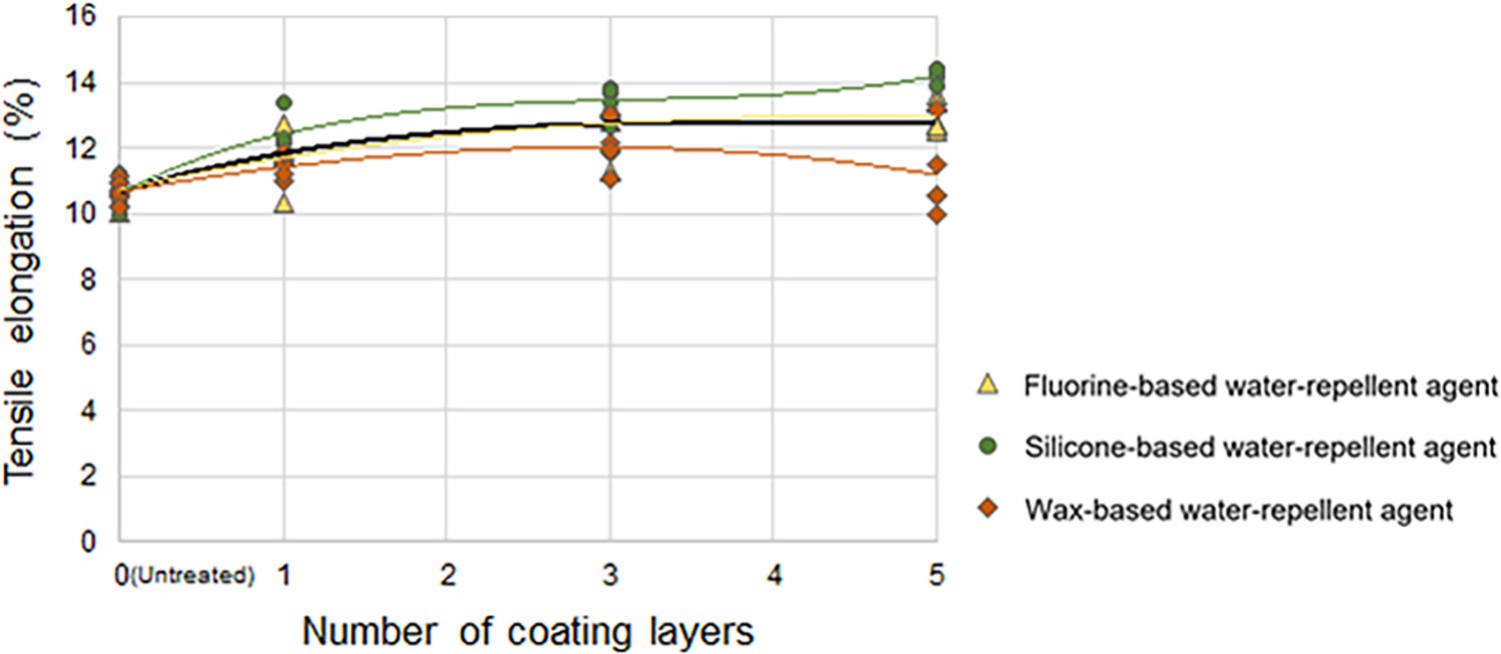
Tensile elongation of the cotton woven fabric according to the number of coating layers of the water-repellent agent. The black line represents the trendline (third-order polynomial).

Fig 7 presents the effects of the number of coating layers of the water-repellent agent on the tensile elongation of the treated cotton woven fabric. For the fluorine- and silicone-based water-repellent agents, the tensile elongation of the treated fabric increased as the number of coating layers increased, indicating its improved stretching compared to the untreated samples (average tensile elongation of 10.67%). In particular, the silicone-based water-repellent agent increased the tensile elongation of the treated fabric (average of 14.20% with five coating layers) more than that of the fluorine-based water-repellent agent (average of 12.98% with five coating layers). Meanwhile, the wax-based water-repellent agent followed the overall trend of the fluorine- and silicone-based water-repellent agents up to three coating layers. However, with five coating layers, the tensile elongation decreased again (with one, three, and five coating layers: 11.42%, 12.01%, and 11.18%, respectively).

#### Changes in stiffness according to the number of coating layers of the water-repellent agent

**Fig 8:**
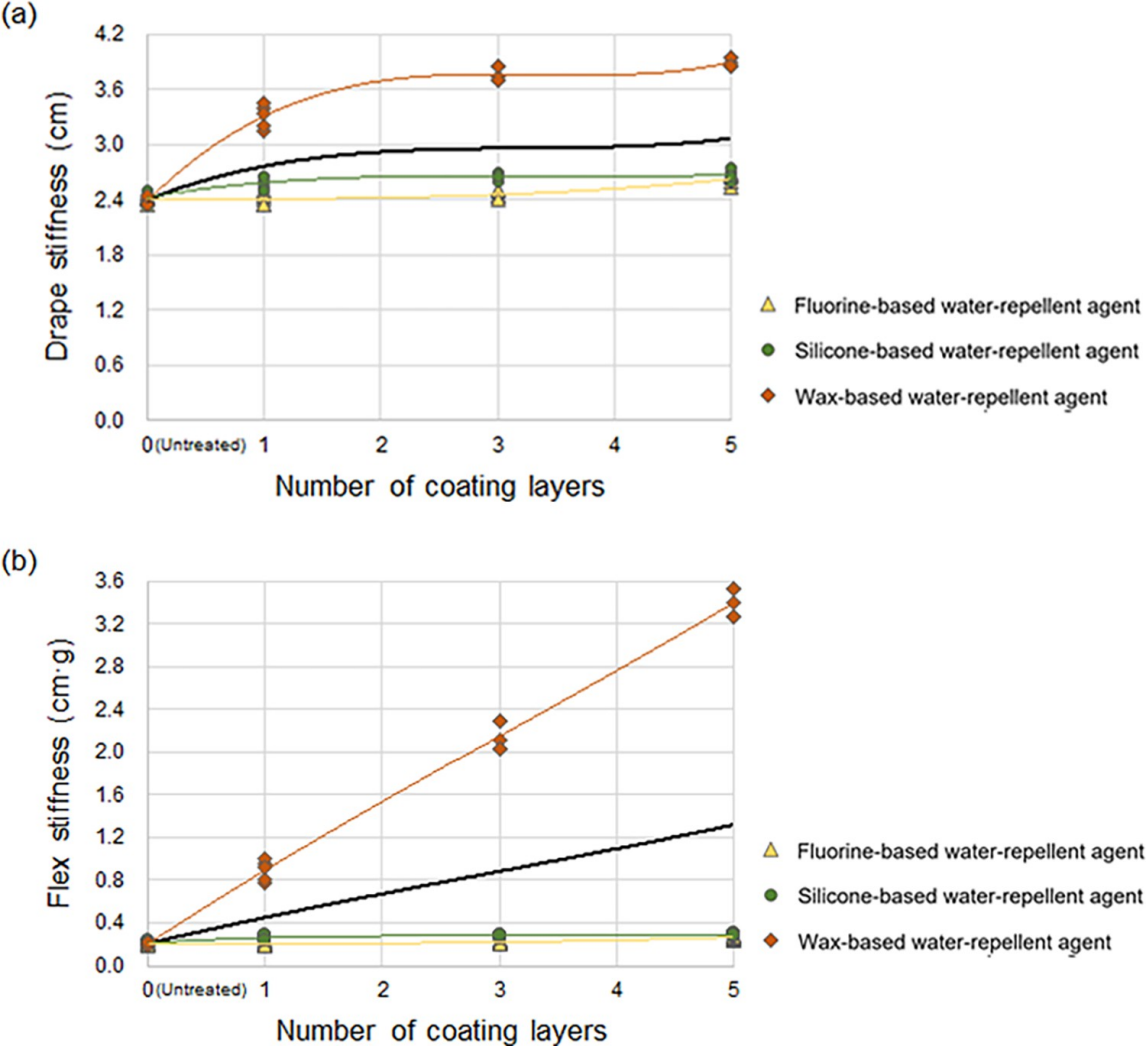
Stiffness of the cotton woven fabrics according to the number of coating layers of the water-repellent agents: (a) drape stiffness and (b) flex stiffness. The black line represents the trendline (third-order polynomial).

Fig 8 presents the effects of the number of coating layers of the water-repellent agent on the stiffness of the treated cotton woven fabric. The overall drape stiffness of the treated fabric increased as the number of coating layers of the water-repellent agent increased, regardless of the type of water-repellent agent, as indicated by the trendline in Fig 8(A). In particular, the wax-based water-repellent agent increased the drape stiffness of the treated fabric significantly more than that of the fluorine- and silicone-based water-repellent agents, which resulted in the higher stiffness (average drape stiffness with no treatment: 2.41 cm; average with one coating layer of the fluorine- and wax-based water-repellent agent: 2.59 and 3.31 cm, respectively). This can be ascribed to the solid state of the wax-based water-repellent agent, which does not easily permeate the treated fabric. For the fluorine- and silicone-based water-repellent agents, the drape stiffness of the treated fabric increased with the number of coating layers with a significantly lower increase in the fabric stiffness than that with the wax-based water-repellent agent. The flex stiffness of the treated cotton woven fabric rapidly increased as the number of coating layers of the wax-based water-repellent agent in solid state increased, as shown in the trendline of Fig 8(B), similar to the drape stiffness. Conversely, even if the number of coating layers of the fluorine- and silicone-based water-repellent agents in liquid state were increased, they were absorbed into the treated fabric, demonstrating flexibility similar to that of the untreated fabric (average flex stiffness with no treatment: 0.21 cm·g; average with five coating layers of the fluorine- and silicone-based water-repellent agent: 0.27 and 0.29 cm·g, respectively).

#### Changes in the water repellency according to the number of coating layers of the water-repellent agents

**Fig 9:**
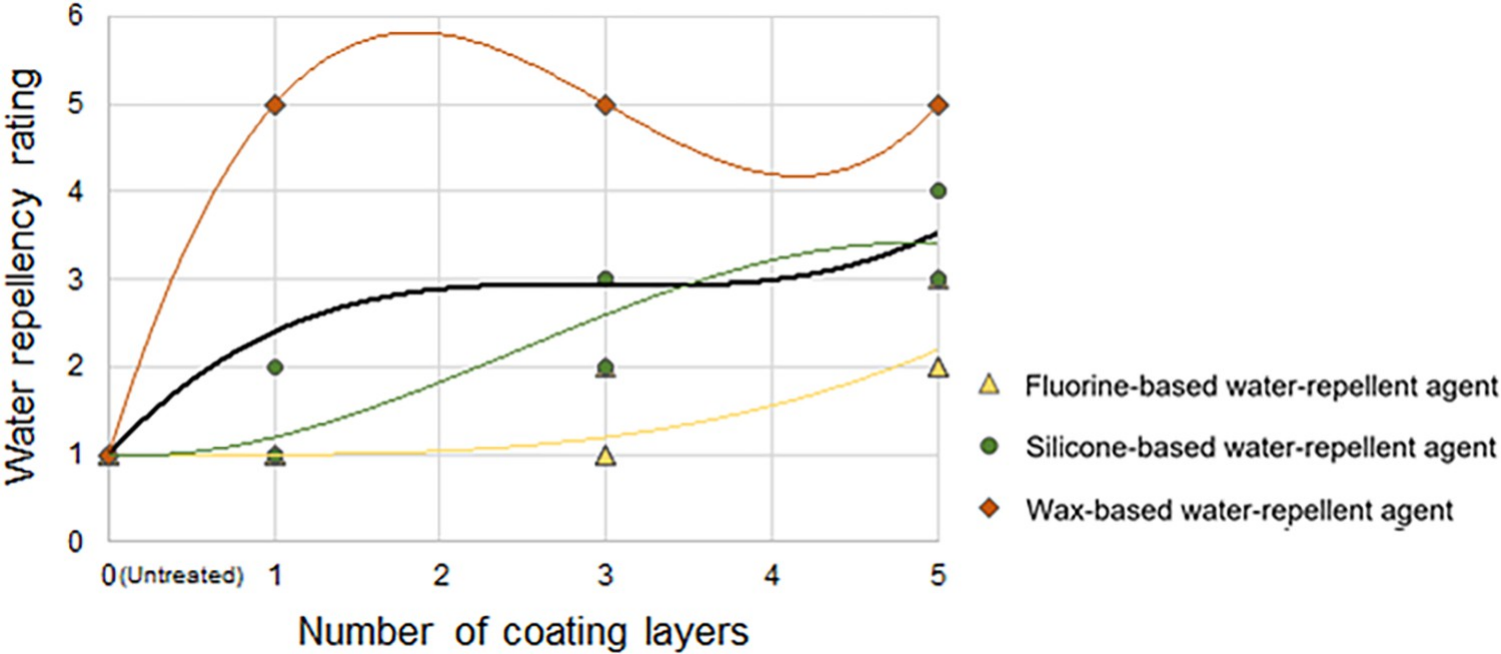
Water repellency of the cotton woven fabric according to the number of coating layers of the water-repellent agent. The black line represents the trendline (third-order polynomial).

Fig 9 presents the changes in the water repellency rating of the treated cotton woven fabric according to the number of coating layers of the water-repellent agents. The untreated cotton woven fabrics exhibited a low water repellency rating of one, as shown in Fig 9. The fluorine- and silicone-based water-repellent agents in liquid state obtained average ratings of 1 and 1.2 with one coating layer, respectively, which indicate minimal water repellency. However, the water repellency of the fabrics treated with the two liquid water-repellent agents gradually increased with repeated coatings. Nevertheless, the fabrics treated with the fluorine-based water-repellent agent still demonstrated a low water repellency rating of 2.2 even with five coating layers. For the silicone-based water-repellent agent, the degree of increase in water repellency with repeated coatings was greater than that of the fluorine-based water-repellent agent having a higher rating of 3.4 with five coating layers. Meanwhile, the solid wax-based water-repellent agent increased the water repellency grade of the treated fabric to a rating of 5 even with only one coating layer, which was maintained in the subsequent coatings. Therefore, for the liquid water-repellent agents, multiple repeated layers, especially five or more layers for the fluorine-based water-repellent agent, are needed to obtain sufficient water repellency. Conversely, one coating layer of the wax-based water-repellent agent is recommended to retain the comfort of the wearer.

## Conclusion

This study aims to investigate the effects of different types of household water-repellent agents that are easily accessible to consumers and number of coating layers on the physical properties of treated cotton woven fabrics. To this end, the physical properties of the fabrics treated once, three times, and five times with the fluorine-, silicone-, and wax-based water-repellent agents were compared. The findings of this study are as follows:

1. The thickness of the treated cotton woven fabric increased with the number of coating layers of the water-repellent agent. Specifically, according to the type of water-repellent agent, the fluorine- and silicone-based water-repellent agents slightly increased the thickness of cotton woven fabric, whereas the wax-based water-repellent agent caused the highest increase in the thickness.
2. The weight of the cotton woven fabric increased with the number of coating layers of the water-repellent agent. According to the type of the water-repellent agent, the weight of the cotton woven fabric increased with the coatings in the following order: wax- > silicone- > fluorine-based water-repellent agents. In particular, the wax-based water-repellent agent demonstrated a faster weight increase with repeated coatings than the other two water-repellent agents.
3. The overall tensile elongation increased with the number of coating layers of the water-repellent agent. The fluorine- and silicone-based water-repellent agents followed this overall tendency. However, for the wax-based water-repellent agent, the elongation increased up to three coating layers and decreased with five coating layers.
4. The drape and flex stiffness increased with the number of coating layers of the water-repellent agent. In particular, the wax-based water-repellent agent greatly increased the drape and flex stiffness of the cotton woven fabrics compared to the silicone- and fluorine-based water-repellent agents.
5. The water repellency, which is the main function of water-repellent agents, of the cotton woven fabrics increased with the number of coating layers of the water-repellent agent. The fluorine-based water-repellent agent did not exhibit high water repellency in the treated fabrics even with five coating layers (average rating of 2.2). However, the wax-based water-repellent agent demonstrated the highest rating of 5 even with one coating layer, which was maintained after five coating layers. Meanwhile, the silicone-based water-repellent agent had a medium water repellency rating of 3.4 with five coating layers.

Therefore, the thickness, weight, and stiffness of the treated cotton woven fabrics, which indicate the discomfort of the wearer, increased with the number of coating layers for the fluorine- and silicone-based water-repellent agents. However, the amount of increase on these properties was minimal. Therefore, multiple coating layers, especially five or more layers for the fluorine-based water-repellent agent, are recommended to obtain excellent water repellency. The wax-based water-repellent agent demonstrated better water repellency than the other two agents. However, considering the comfort of the wearer, only one coating layer is recommended because the agent considerably increased the thickness, weight, and stiffness of the treated fabrics. In addition, the wax-based water-repellent agent is recommended if water repellency is prioritized over comfort, whereas the silicone-based water-repellent agent is recommended if comfort is prioritized over water repellency. The results of this study are expected to provide consumers with the characteristics of household water-repellent agents according to their composition, and optimal conditions for using each agent, thereby strengthening and supplementing the water-repellent fabric industry. Follow-up research on the subjective comfort of wearers based on the type of water-repellent agents and number of coating layers could provide more practical information to consumers and the water repellency industry.

## Supporting information

S1 Table Physical properties of cotton woven fabrics measured five times. (PDF)
