# Supplementary material for: Effects of household water-repellent agents and number of coating layers on the physical properties of cotton woven fabrics
Source: PLoS One. 2023 Apr 14;18(4):e0283261. doi: 10.1371/journal.pone.0283261 (PMC10104306; doi:10.1371/journal.pone.0283261)
Supplement: S1 Table — (PDF) [file pone.0283261.s001.pdf]

S1 Table. Physical properties of cotton woven fabrics measured five times.

Sample name	Water-repellent agent	Number of coating layers	Thickness (mm)	Weight (g/m$^{2)}$	Tensile strength	Tensile elongation (%)	Drape stiffness (cm)	Flex stiffness (cm·g)	Water repellency
UT	Fluorine-based	0	0.33	148.20	32.90	10.97	2.40	0.21	1
UT	Fluorine-based	0	0.35	148.50	30.84	10.03	2.40	0.21	1
UT	Fluorine-based	0	0.34	148.20	28.96	10.51	2.45	0.22	1
UT	Fluorine-based	0	0.33	146.70	30.74	10.87	2.40	0.21	1
UT	Fluorine-based	0	0.33	147.70	32.84	11.23	2.35	0.19	1
F-1	Fluorine-based	1	0.36	146.90	29.58	12.76	2.35	0.19	1
F-1	Fluorine-based	1	0.37	146.10	30.84	11.92	2.40	0.20	1
F-1	Fluorine-based	1	0.36	145.10	30.70	11.69	2.50	0.23	1
F-1	Fluorine-based	1	0.36	145.30	31.74	11.74	2.45	0.22	1
F-1	Fluorine-based	1	0.36	144.80	28.64	10.37	2.35	0.19	1
F-3	Fluorine-based	3	0.37	147.50	28.94	11.29	2.45	0.22	1
F-3	Fluorine-based	3	0.38	145.20	30.68	12.78	2.50	0.23	1
F-3	Fluorine-based	3	0.37	147.40	32.70	13.26	2.45	0.22	1
F-3	Fluorine-based	3	0.38	147.90	30.62	13.19	2.50	0.23	2
F-3	Fluorine-based	3	0.38	146.80	31.42	13.23	2.40	0.20	1
F-5	Fluorine-based	5	0.38	146.80	31.88	12.75	2.70	0.29	2
F-5	Fluorine-based	5	0.38	146.40	28.76	12.49	2.55	0.24	2
F-5	Fluorine-based	5	0.38	148.10	31.80	12.66	2.65	0.27	2
F-5	Fluorine-based	5	0.38	145.90	32.16	13.6	2.60	0.26	2
F-5	Fluorine-based	5	0.38	147.00	32.16	13.38	2.65	0.27	3
UT	Silicone-based	0	0.34	148.00	29.62	10.92	2.35	0.19	1
UT	Silicone-based	0	0.33	147.80	31.76	11.19	2.45	0.22	1
UT	Silicone-based	0	0.34	147.50	31.90	10.77	2.50	0.23	1
UT	Silicone-based	0	0.33	148.30	31.54	9.99	2.45	0.22	1
UT	Silicone-based	0	0.35	147.90	30.94	10.29	2.40	0.21	1
S-1	Silicone-based	1	0.37	148.80	32.28	12.13	2.60	0.26	1
S-1	Silicone-based	1	0.36	148.20	29.66	12.13	2.65	0.28	1
S-1	Silicone-based	1	0.36	147.60	32.40	12.27	2.55	0.25	2
S-1	Silicone-based	1	0.36	147.20	31.32	12.22	2.65	0.28	1

S-1	Silicone-based	1	0.35	147.30	33.50	13.4	2.50	0.23	1
S-3	Silicone-based	3	0.37	149.20	32.66	13.36	2.65	0.28	3
S-3	Silicone-based	3	0.38	145.60	30.50	12.63	2.70	0.29	2
S-3	Silicone-based	3	0.38	147.70	33.42	13.69	2.70	0.29	3
S-3	Silicone-based	3	0.38	147.70	32.32	13.81	2.60	0.26	2
S-3	Silicone-based	3	0.38	147.40	32.52	13.77	2.65	0.28	3
S-5	Silicone-based	5	0.38	150.00	31.16	14.18	2.70	0.30	3
S-5	Silicone-based	5	0.38	153.20	33.66	14.37	2.60	0.27	3
S-5	Silicone-based	5	0.38	150.60	30.06	14.18	2.70	0.30	4
S-5	Silicone-based	5	0.38	151.90	33.66	14.39	2.75	0.31	3
S-5	Silicone-based	5	0.38	150.50	32.68	13.87	2.65	0.28	4
UT	Wax-based	0	0.35	146.40	30.56	11.15	2.35	0.19	1
UT	Wax-based	0	0.33	148.50	30.13	10.45	2.40	0.21	1
UT	Wax-based	0	0.34	148.20	31.78	10.89	2.40	0.21	1
UT	Wax-based	0	0.32	147.20	30.98	10.62	2.35	0.19	1
UT	Wax-based	0	0.33	146.90	31.48	10.19	2.45	0.22	1
W-1	Wax-based	1	0.38	243.00	27.28	10.99	3.15	0.77	5
W-1	Wax-based	1	0.38	245.80	31.52	11.57	3.40	0.96	5
W-1	Wax-based	1	0.38	246.10	31.46	11.47	3.45	1.01	5
W-1	Wax-based	1	0.38	246.80	28.46	11.2	3.20	0.80	5
W-1	Wax-based	1	0.39	241.40	31.14	11.85	3.35	0.92	5
W-3	Wax-based	3	0.44	427.10	32.66	11.07	3.75	2.12	5
W-3	Wax-based	3	0.44	430.20	32.50	11.87	3.70	2.03	5
W-3	Wax-based	3	0.42	359.60	28.04	12.15	3.85	2.29	5
W-3	Wax-based	3	0.41	430.60	32.40	11.92	3.85	2.29	5
W-3	Wax-based	3	0.45	359.90	28.10	13.05	3.70	2.03	5
W-5	Wax-based	5	0.75	600.00	30.28	11.53	3.85	3.27	5
W-5	Wax-based	5	0.65	582.90	32.22	13.19	3.90	3.40	5
W-5	Wax-based	5	0.59	528.20	31.44	10.58	3.95	3.53	5
W-5	Wax-based	5	0.68	574.50	31.66	10.01	3.95	3.53	5
W-5	Wax-based	5	0.65	578.20	31.84	10.58	3.85	3.27	5
